# Water Extract of Mungbean (*Vigna radiata* L.) Inhibits Protein Tyrosine Phosphatase-1B in Insulin-Resistant HepG2 Cells

**DOI:** 10.3390/molecules26051452

**Published:** 2021-03-07

**Authors:** Orathai Saeting, Kasemsiri Chandarajoti, Angsuma Phongphisutthinan, Parichat Hongsprabhas, Sudathip Sae-tan

**Affiliations:** 1 Department of Food Science and Technology, Faculty of Agro-Industry, Kasetsart University, Bangkok 10900, Thailand; orathai.saeting@gmail.com (O.S.); parichat.h@ku.ac.th (P.H.); 2 Department of Pharmaceutical Chemistry, Faculty of Pharmaceutical Sciences, Prince of Songkla University, Hat-Yai, Songkhla 90112, Thailand; kasemsiri@pharmacy.psu.ac.th; 3 Drug Delivery System Excellence Center, Faculty of Pharmaceutical Sciences, Prince of Songkla University, Hat-Yai, Songkhla 90112, Thailand; 4 Division of Pharmaceutical Sciences, Faculty of Pharmacy, Thammasat University, Rangsit Center, Pathumthani 12121, Thailand; angsumaw@tu.ac.th

**Keywords:** diabetes, glucose uptake, mungbean, protein tyrosine phosphatase-1B

## Abstract

The present study aimed to investigate the effects of mungbean water extract (MWE) on insulin downstream signaling in insulin-resistant HepG2 cells. Whole seed mungbean was extracted using boiling water, mimicking a traditional cooking method. Vitexin and isovitexin were identified in MWE. The results showed that MWE inhibited protein tyrosine phosphatase (PTP)-1B (IC_50_ = 10 μg/mL), a negative regulator of insulin signaling. MWE enhanced cellular glucose uptake and altered expression of genes involved in glucose metabolism, including forkhead box O1 (FOXO1), phosphoenolpyruvate carboxykinase (PEPCK), and glycogen synthase kinase (GSK)-3β in the insulin-resistant HepG2 cells. In addition, MWE inhibited both α-amylase (IC_50_ = 36.65 mg/mL) and α-glucosidase (IC_50_ = 3.07 mg/mL). MWE also inhibited the formation of advanced glycation end products (AGEs) (IC_50_ = 2.28 mg/mL). This is the first study to show that mungbean water extract increased cellular glucose uptake and improved insulin sensitivity of insulin-resistant HepG2 cells through PTP-1B inhibition and modulating the expression of genes related to glucose metabolism. This suggests that mungbean water extract has the potential to be a functional ingredient for diabetes.

## 1. Introduction

Diabetes is a metabolic disorder with various complications [1]. More than 90% of diabetic patients are with type 2 diabetes clinically presented with insulin resistance [2]. It has been projected to increase by 25% in 2030 and 51% in 2045 [3]. A prominent feature of diabetes is hyperglycemia due to abnormal carbohydrate metabolism. The chronic high blood sugar level in diabetes causes organ damage, including eyes, kidneys, nerves, heart, and blood vessels [4]. One of the multiple therapeutic approaches to manage hyperglycemia is inhibiting carbohydrate metabolizing enzymes, i.e., α-amylase and α-glucosidase [5,6]. Interfering carbohydrate digestion results in reducing the increased postprandial blood glucose. Drugs display such mechanism include acarbose and miglitol [7]. However, side effects such as flatulence, diarrhea, liver disorder and abdominal distention were reported [8]. The unwanted side effects can cause patient incompliance and poor treatment effectiveness. 

A complex system, including insulin function, regulates the homeostasis of blood glucose. The liver mainly regulates glucose level by altering the levels of hepatic glucose release through the *de novo* glucose production process (gluconeogenesis) and glycogen breakdown (glycogenolysis) [9,10]. Insulin resistance is a hallmark of type 2 diabetes, causing irresponsive insulin signaling in peripheral tissues. In hepatocytes, insulin binds insulin receptors, transduces phosphorylation of insulin receptor substrate, which then activates phosphatidylinositol 3-kinase (PI-3K) for the subsequent phosphorylation of protein kinase B (PKB; also called PKB/Akt) [11]. Protein tyrosine phosphatase-1B (PTP-1B) is a negative regulator of insulin signal transduction. PTP-1B regulates a downstream signal of insulin [11,12]. PTP-1B diminishes insulin signaling by dephosphorylating insulin receptors, resulting in the down-regulation of the insulin signaling pathway and glucose uptake reduction [11,12]. Dephosphorylation of insulin receptors causes the inactivation of PI-3K and PKB/Akt, leading to the impaired glucose metabolism [11]. Therefore, inhibition of PTP-1B reverts clinical consequences generated by insulin resistance and PTP-1B has been identified as a potential target and extensively studied in diabetic management.

Inactivation of the PI-3K/Akt pathway, a downstream insulin signaling, leads to the loss of insulin signaling in hepatocytes and severe insulin resistance, resulting in uncontrolled glucose production and glycogen synthesis in part through alteration of mRNA expression [10,11]. PI-3K/Akt cascade regulates fasting gene expression mediators, including foxhead box O1 (FOXO1), a transcription factor regulating genes that control glucose production [2,13]. A previous study reported that inhibition of FOXO1 improved hepatic metabolism during insulin resistance [13]. FOXO1 also regulates phosphoenolpyruvate carboxykinase (PEPCK), a rate-limiting enzyme of gluconeogenesis [9]. Another targeted gene in the Akt pathway is glycogen synthase kinase (GSK)-3β, an inhibitor of glycogen synthesis [11]. Therefore, the regulation of glucose uptake, glucose production, and glycogen synthesis are crucial in controlling glucose homeostasis. The end-stage diabetic patients are those who have the disease’s complications leading to morbidity and mortality. A complex cascade of complications involves a prolongation of elevated plasma glucose levels [14]. Blood glucose can covalently adduct to plasma proteins through a non-enzymatic reaction known as glycation. Protein glycation leads to the formation of advanced glycation end products (AGEs). Accumulation of AGEs is significantly involved in the pathogenesis of various diabetic complications [15]. Therefore, inhibition of the formation of AGEs is an approach to reduce diabetic complications and mortality of diabetic patients.

Mungbean has been reported for its biological activities, including angiotensin I-converting enzyme (ACE) inhibition [16], efficiency to reduce lipid accumulation [17] and inflammation [17,18,19], anti-formation of advanced glycation end products (AGEs) [15], and anti-diabetes activities [20,21]. Mungbean has been recommended as an alternative to diets for diabetic patients for improving blood sugar profile. Mungbean soup beverage is considered a healthy drink for diabetic patients [21]. Previous studies identified two phenolic isomers, including vitexin and isovitexin, from the ethanolic and methanolic mungbean extract [15,22]. The isomers were mainly extracted from the mungbean seed coat [21,23]. The ethanolic seed coat extract showed α-glucosidase inhibition [20]. Anti-diabetic activity of the ethanolic seed coat extract was investigated in KK-Ay mice [21] and type 2 diabetic *db/db* mice [20]. The results also indicated that the ethanolic seed coat extract reduced blood glycated hemoglobin and serum glucose levels as well as improved both glucose tolerance and insulin response to glucose metabolism activities. 

Although previous studies showed the effects of ethanolic and methanolic mungbean extract in lowering blood sugar [17,21], none of the studies investigated downstream mechanisms involved in insulin signaling. Studies of mungbean water extraction are rarely available, and only antioxidative effects properties were reported [24,25], thus lacking in anti-diabetic effects. This study employed water and temperature for mungbean extraction, which is a general cooking method for mungbean soup. We investigated the effects of MWE on insulin signaling and gene expression involved in glucose metabolism.

## 2. Results

### 2.1. Total Phenolic Content and Flavonoid Identification of MWE 

**Figure 1:**
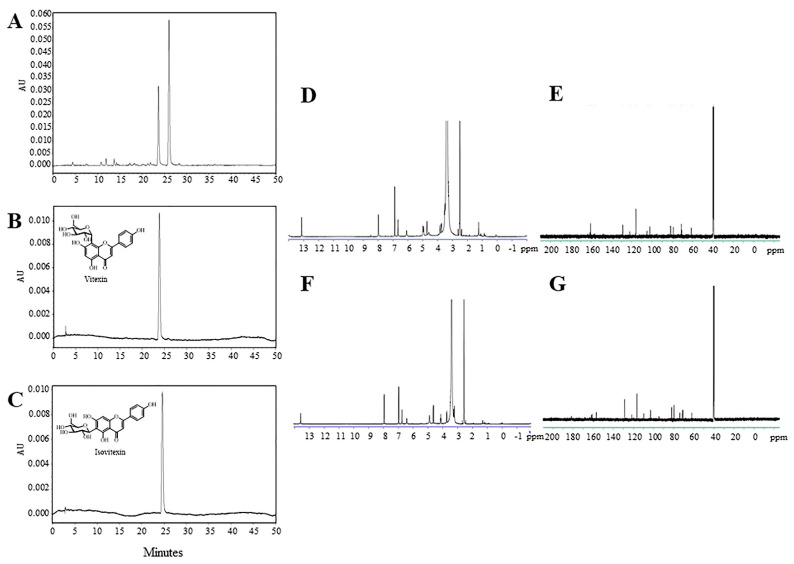
Representative high-performance liquid chromatography equipped with diode array detector (HPLC-DAD) chromatographic profiles of (**A**) mungbean water extract (MWE), (**B**,**C**) collected fractions 1 and 2 from a semi-preparative HPLC (λ = 337 nm) identified against standards vitexin and isovitexin, respectively. Nuclear magnetic resonance (NMR) spectrum of separated first fraction; (**D**) ^1^H, (**E**) ^13^C-NMR spectrum of separated second fraction; (**F**) ^1^H and (**G**) ^13^C. AU is an arbitrary unit. ppm is part per million.

Mungbean was extracted with boiling water and freeze-dried to obtain mungbean water extract (MWE). The percentage yield of MWE was 3.00 ± 0.18 (n = 6). We determined the phenolic content of MWE in a unit of gallic acid equivalents (GAE) per gram dry weight (DW). The results showed that MWE contained 60.37 mg GAE/g DW. Phenolic isomers were identified by high-performance liquid chromatography (HPLC). Comparing to chromatograms of vitexin and isovitexin standards, the chromatogram of MWE at 337 nm showed two major compounds and were identified as vitexin (R_t_ = 23.8 min) and isovitexin (R_t_ = 26.2 min), respectively (Figure 1A). We prepared a large batch size of MWE and used semi-preparative HPLC to separate the isomeric compounds (as shown in Figure S1 and referred to fraction 1 and fraction 2) for further nuclear magnetic resonance (NMR) analyses. The individual HPLC chromatograms of fraction 1 and fraction 2 were shown in Figure 1B and Figure 1C. ^1^H and ^13^C NMR spectra of each fraction from semi-preparative HPLC correspond with the structure elucidation results of vitexin and isovitexin in the literature [15,24]. This result confirmed that the chromatograms shown in Figure 1B,C were vitexin and isovitexin (^1^H: Figure 1D,F, ^13^C: Figure 1E,G).

Vitexin: yellow powder ^1^H NMR (DMSO-*d_6_*, 600 MHz) δ: 13.15 (1H, s), 7.98 (2H, d, *J* = 8.7 Hz), 6.88 (2H, d, *J* = 8.7 Hz), 6.65 (1H, s), 6.08 (1 H, s), 4.96 (2H, br), 4.71 (1H, d, *J* = 9.8 Hz) 3.90–3.20 (4H, m). ^13^C NMR (DMSO-*d_6_*, 150 MHz) δ: 182.2, 164.0, 162.7, 161.1, 160.4, 156.1, 128.7, 121.85, 115.8, 104.8, 104.2, 102.1, 98.3, 81.7, 78.8, 73.5, 71.1, 70.6, 61.4.

Isovitexin: yellow powder. ^1^H NMR (DMSO-*d_6_*, 600 MHz) δ: 13.57 (1H, s), 7.95 (2H, d, *J* = 8.8 Hz), 6.97 (2H, d, *J* = 8.8 Hz), 6.75 (1H, s), 6.42 (1H, s), 4.63 (1H, d, *J* = 9.8 Hz,), 4.13 (1H, m), 3.73 (1H, d, *J* = 11.7 Hz) 3.60–3.20 (4H, m). ^13^C NMR (DMSO-*d_6_*, 150 MHz) δ: 181.9, 163.5, 163.2, 161.7, 160.1, 156.9, 128.7, 121.6, 116.4, 109.6, 103.4, 102.8, 93.6, 81.8, 79.5, 73.8, 71.1, 70.7, 61.9.

NMR results confirmed that boiling water could extract vitexin and isovitexin from whole mungbean seed as same as methanol and ethanol [17,21,23] and that boiling water did not affect the stability of vitexin and isovitexin. To standardize MWE, we prepared three individual batches of MWE and determined vitexin and isovitexin contents. The extracted vitexin and isovitexin from MWE were quantified using standard curves (5–50 μg/mL). The standardized MWE showed the amount of vitexin and isovitexin at a ratio of 1 to 2 (9.37 and 17.98 mg/g DW, respectively). 

### 2.2. Inhibitory Effects of MWE against A-Amylase and A-Glucosidase

**Figure 2:**
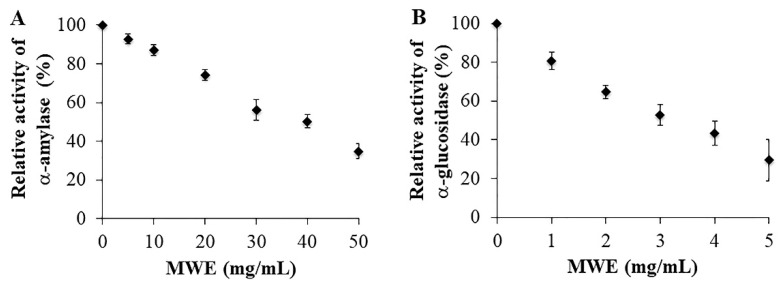
Inhibitory activities of mungbean water extract (MWE) on (**A**) α-amylase and (**B**) α-glucosidase. Values are expressed as mean ± SD (n = 3). IC_50_ values were determined using GraphPad Prism 7 statistical package.

A-Amylase and A-Glucosidase Are Enzymes That Digest Carbohydrates to monosaccharides for further absorption into the bloodstream. Inhibition of these enzymes is an effective approach to decrease postprandial blood sugar in diabetic patients [5]. We determined the inhibitory effects by using an isolated α-amylase from porcine and an α-glucosidase from *Saccharomyces cerevisiae*. The activities of both enzymes incubated with different concentrations of MWE were shown in Figure 2. The results showed that MWE inhibited α-amylase and α-glucosidase in a dose-dependent manner with IC_50_ values of 36.65 mg/mL and 3.07 mg/mL, respectively (Figure 2A,B). In the present study, acarbose, a synthetic drug used in diabetic treatment, was used as a positive control. Acarbose inhibited α-amylase and α-glucosidase with an IC_50_ value of 0.01 mg/mL and 6.92 mg/mL, respectively (Figure S2).

### 2.3. Inhibitory Effects of MWE against AGEs Formation

**Figure 3:**
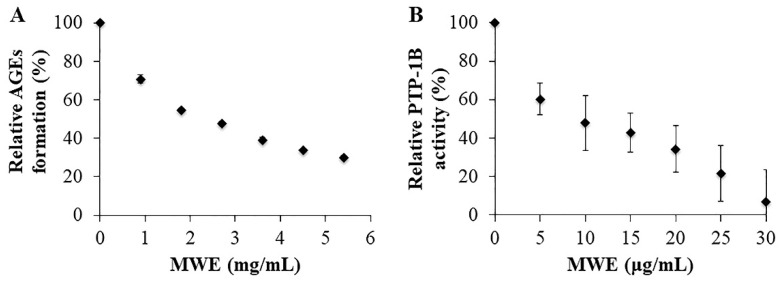
Inhibitory activities of mungbean water extract (MWE) on (**A**) advanced glycation end products (AGEs) formation and (**B**) protein tyrosine phosphatase-1B (PTP-1B) activity. Values are expressed as the mean ± SD (n = 3). IC_50_ values were determined using GraphPad Prism 7 statistical package.

To assess the anti-glycation effects of MWE, the bovine serum albumin (BSA)-glucose model was adopted in the present study. Here, we found that MWE (0.9–5.4 mg/mL) inhibited the formation of AGEs in a dose-dependent manner with an IC_50_ value of 2.28 mg/mL (Figure 3A). Aminoguanidine (AG), the first discovered AGEs formation inhibitor, was used as a positive control in this study (IC_50_ = 0.63 mg/mL) (Figure S3). 

### 2.4. Inhibitory Effects of MWE against PTP-1B

PTP-1B serves as a therapeutic target for type 2 diabetes by improving insulin signaling. We found that MWE (5–30 μg/mL) inhibited PTP-1B in a dose-dependent manner with an IC_50_ value of 10 μg/mL (Figure 3B). This result suggested that MWE can improve insulin signaling in insulin-resistant HepG2 cells. Sodium orthovanadate (NaVO_4_), a known inhibitor of protein tyrosine phosphatases and used for diabetic patients [25,26], was used as a positive control in this study (IC_50_ = 0.65 mg/mL) (Figure S3).

### 2.5. The Effect of MWE on Cytotoxicity at 24 H Of Treatment in Hepg2 Cells

**Figure 4:**
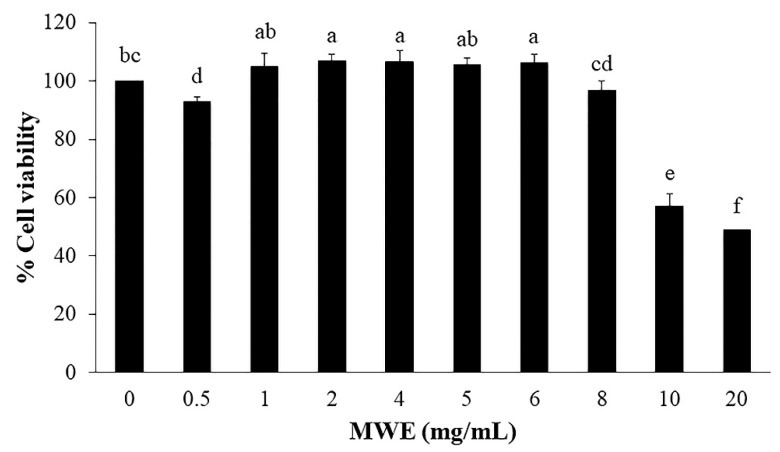
Cytotoxicity of mungbean water extract (MWE) on the survival of HepG2 cells at different concentrations. Values are expressed as the mean ± SD (n = 3). Statistical analyses were performed using Duncan’s multiple range test one-way analysis of variance (ANOVA). Different letters (a–f) indicate statistical differences (*p* < 0.05).

To further investigate the cellular mechanisms of MWE. We used HepG2 cells as a model since the liver is the main organ to regulate the glucose level. The cytotoxicity of MWE towards HepG2 cells was assayed after 24 h of treatment with increasing concentration of MWE (Figure 4). The results showed the absence of significant cytotoxicity of the MWE in the range of 0.5–8 mg/mL according to 3-(4,5-dimethylthiazol-2-yl)-2,5-diphenyltetrazolium bromide (MTT) assay. Therefore, this range of MWE was used for further experiments.

### 2.6. The Effect of MWE on Cellular Glucose Uptake of Insulin-Resistant Hepg2 Cells

**Figure 5:**
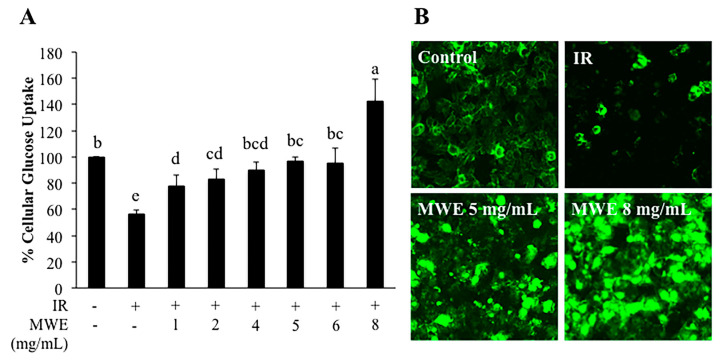
Cellular glucose uptake in the control cells and the insulin-resistant HepG2 cells without and with mungbean water extract (MWE) treatment. (**A**) Percentage of cellular glucose uptake of the control cells and the insulin-resistant HepG2 cells without and with MWE. (**B**) Fluorescence intensity of Deoxy-2-[(7-nitro-2,1,3-benzoxadiazol-4-yl)amino]-D-glucose (2-NBDG) in the control cells and the insulin-resistant HepG2 cells without and with MWE. Statistical analyses were performed using Duncan’s multiple range test one-way analysis of variance (ANOVA). Values are expressed as the mean ± SD (n = 3). Different letters (a–e) indicate statistical differences (*p* < 0.05). IR is insulin-resistant HepG2 cells.

To further investigate the effects of MWE on glucose uptake in insulin resistance, we used insulin-resistant cells. We proposed that the inhibitory effects of MWE against PTP-1B improved insulin signaling of insulin-resistant cells, which led to the increase of cellular glucose uptake corresponding to the reduction of blood glucose in diabetic mice in previous studies [20,21]. Insulin-resistant HepG2 cells were used to further investigate the improvement of insulin signaling. We demonstrated that insulin-resistant HepG2 cells presented a significantly lower percentage of glucose uptake than the control cells (Figure 5A). Treatment of MWE (1–8 mg/mL) significantly increased glucose uptake 38.2–152.1% (Figure 5A) as reflected by fluorescence intensity (Figure 5B). 

### 2.7. The Effect of MWE on the Expression of Genes Related to Glucose Metabolism in Insulin-Resistant Hepg2 Cells

**Figure 6:**
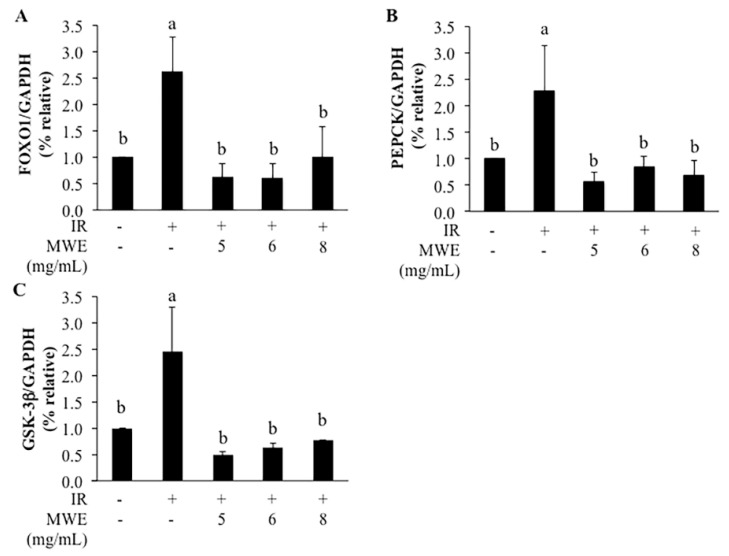
Percentage relative mRNA expression of genes related to glucose metabolism in insulin-resistant HepG2 cells without and with mungbean water extract (MWE) (**A**) forkhead box O1 (FOXO1) (**B**) phosphoenolpyruvate carboxykinase (PEPCK) (**C**) glycogen synthase kinase-3β (GSK-3β). mRNA of target genes was relative to glyceraldehyde 3-phosphate dehydrogenase (GAPDH). Values are expressed as the mean ± SD (n = 3). Statistical analyses were performed using Duncan’s multiple range test one-way analysis of variance (ANOVA). Different letters (a–b) indicate statistical differences (*p* < 0.05). IR is insulin-resistant HepG2 cells.

Furthermore, we investigated the expression of genes related to glucose metabolism in insulin-resistant HepG2 cells to confirm the effects of MWE on glucose metabolism. Gluconeogenesis and glycogen synthesis in the liver is regulated by glucose level. FOXO1 is the transcription factor, which regulates PEPCK, a rate-limiting enzyme of gluconeogenesis [9,10]. Our results showed that expressions of FOXO1 and PEPCK were significantly increased in insulin-resistant HepG2 cells, indicating the upregulated glucose production in insulin-resistant hepatocytes. The increases of FOXO1 and PEPCK expression in insulin-resistant HepG2 cells were significantly lowered after MWE treatment (Figure 6A,B). This indicated that MWE treatment reduced glucose production in insulin-resistant HepG2 cells. GSK-3β is an enzyme regulating glycogen synthesis in response to insulin [11]. The expression of GSK-3β in insulin-resistant HepG2 cells was significantly higher than the control cells. We also found that MWE treatment significantly reduced the expression of GSK-3β compared to insulin-resistant HepG2 cells (Figure 6C). This indicated MWE treatment reduced the inhibition of glycogen synthesis.

## 3. Discussion

In the present study, mungbean was extracted using boiling water, mimicking a traditional cooking method. The results showed that boiling water was able to extract both vitexin and isovitexin from mungbean (Figure 1 and Figure S1), similar to those found in the ethanolic and methanolic extract [15,22]. In the present study, MWE showed inhibitory effects on both α-amylase and α-glucosidase. The results are consistent with previous studies demonstrating that ethanolic mungbean seed coat extract inhibited α-glucosidase [20], and polyphenols extracted by alkaline hydrolysis inhibited α-amylase and α-glucosidase [27]. Interestingly, we found that MWE was more efficient in inhibiting α-glucosidase but less efficient in inhibiting α-amylase compared to acarbose. This suggested the advantage of MWE because the lower effect of MWE in α-glucosidase inhibition results in the remaining α-glucosidase, which can digest polysaccharides, which minimizes uncomfortable abdominal symptoms from the use of acarbose in diabetic patients.

In prolonged hyperglycemia conditions, blood glucose covalently adducts to plasma proteins, leading to the formation of AGEs, which play a dominant role in diabetic complications [15]. Therefore, inhibition of AGEs formation is an approach to reduce the mortality rate of diabetic patients. In this study, we found that MWE exhibited inhibitory effects on AGEs formation. Comparing with other species of beans, the inhibitory effects of mungbean against AGEs formation were more effective than black beans, soybeans, and long beans due to the higher amount of vitexin and isovitexin [15]. Clinical research found that AG caused side effects, including gastrointestinal symptoms, abnormalities in liver function, flu-like symptoms, and anti-neutrophil cytoplasmic antibody-associated vasculitis [28]. In addition, the use of AG raised attention due to the development of kidney tumors in AG-treated diabetic rats [29]. Although the efficacy of MWE to inhibit AGEs formation was to a lesser extent than that of AG and the safety profile of MWE remains to be investigated; in vivo studies showed that ethanolic mungbean extract was safe in the range of 1–3 g/kg/day [17,20,21]. This may imply the safety of MWE, considered as food. Our results demonstrated that MWE can be a beneficial alternative in lowering AGEs formation.

Inhibition of PTP-1B was proposed to be a therapeutic target for improving insulin signaling by increasing glucose uptake. PTP-1B knockout mice showed increased systematic insulin sensitivity and enhanced glucose tolerance [30]. Liver-specific PTP-1B knockout mice also showed a marked increase in insulin sensitivity [31]. A recent study also showed that inhibition of PTP-1B expression by (−)-epigallocatechin-3-gallate (EGCG) activated insulin signaling resulted in the increase of cellular glucose uptake [32]. The present study showed for the first time that MWE inhibited PTP-1B activity (Figure 3B). The present result is consistent with the previous study showing that *C*-glycosylated derivatives of apigenin, including vitexin and isovitexin, inhibited PTP-1B [33]. The inhibitory effect of MWE against PTP-1B suggested that MWE can improve insulin signaling resulted in an increase in glucose uptake. The result showed that in the presence of MWE, glucose uptake in insulin-resistant HepG2 cells increased. Hence, PTP-1B is a promising target in modifying insulin resistance.

A previous study reported that cocoa flavonoids improved insulin signaling of high glucose-induced insulin signaling blockade in HepG2 cells and modulated glucose uptake and production [34]. Here, we found that MWE lowered the elevated expression of FOXO1, PEPCK, and GSK-3β in insulin-resistant HepG2 cells. These results indicated that MWE contributed to decreased gluconeogenesis and increased glycogen synthesis in the insulin-resistant HepG2 cells.

Collectively, this is the first report demonstrating that MWE is linked to the increased glucose uptake and improved insulin signaling, potentially involving in PTP-1B inhibition. The improvement of insulin signaling by MWE downregulated genes in gluconeogenesis and upregulated a gene in glycogen synthesis in insulin-resistant HepG2 cells. However, further study is needed to thoroughly explore the mechanisms of action of MWE. MWE also showed inhibitory effects against α-amylase and α-glucosidase and AGEs formation. All of these results may be partly due to the presence of vitexin and isovitexin in MWE. However, other components in MWE extracted with water, such as soluble protein, soluble fiber, may also contribute to insulin signaling, thus remaining to be investigated. Besides, the presence of vitexin and isovitexin also confirmed that boiling water was able to extract both isomers similar to that of organic solvent. Thus, water extraction can be utilized to obtain mungbean extract, suggesting a non-hazardous approach for daily human consumption owing to the non-organic solvent involved and environmental friendly for industries to manufacture MWE.

## 4. Materials and Methods

### 4.1. Materials and Reagents

Mungbean, Chainat 84–1 variety, was gifted from Chainat Field Crop Research Center, Chainat, Thailand. They were harvested in September 2015. Vitexin, isovitexin, α -amylase, α-glucosidase, PTP-1B, para-nirophenyl-α-d-glucopyranoside (pNPG), and p-nitrophenyl phosphate (pNPP) were purchased from Sigma Aldrich (St. Louis, MO, USA). 2-Deoxy-2-[(7-nitro-2,1,3-benzoxadiazol-4-yl)amino]-D-glucose (2-NBDG) was purchased from Invitrogen (Waltham, CA, USA). Insulin was purchased from Gibco (Grand Island, NY, USA). Acarbose and dinitrosalicylic acid were purchased from Tokyo Chemical Industry (Tokyo, Japan). Dulbecco’s modified Eagle’s medium (DMEM) was purchased from Hyclone (Logan, UT, USA). Fetal bovine serum and penicillin/streptomycin were purchased from Gibco (Paisley, UK). Total RNA Mini Kit was purchased from Geneaid (New Taipei city, Taiwan). RevertAid First Strand cDNA synthesis kit was purchased from Thermofisher (Vilnus, Lithuania). GeneJET RNA Purification Kit and DNase I, RNase-free were purchased from Thermo Scientific (Vilnus, Lithuania). SYBR Green Supermix was purchased from Bio-rad (California, CA, USA). All other chemicals were of analytical grade and obtained from reputable suppliers.

### 4.2. Preparation of Mungbean Water Extract (MWE)

Water extract of mungbean was prepared according to a traditional cooking method. Briefly, mungbean seeds were cleaned and rinsed with tap water. Fifty grams of the whole bean was put in 500 mL of boiling deionized water and boiled for 20 min, then filtrated through a filter paper (Whatman No.1). The filtrate was collected and evaporated with a rotary evaporator at 150 rpm, 50 °C for 40–50 min to get concentrated extract. The extract was centrifuged at 3500 rpm for 30 min, then freeze-dried. The freeze-dried sample, mungbean water extract (MWE), was kept at −80 °C for further analysis.

### 4.3. Determination of Total Phenolic Content

Total phenolic content in MWE was determined using the Folin–Ciocalteu method [23]. Briefly, 0.1 mL of 5 mg/mL MWE was added to 2.5 mL of distilled water, and 0.25 mL of Folin-Ciocalteu reagent was added and equilibrated for 5 min. Then, the mixture was neutralized with 0.75 mL of 20% Na_2_CO_3_ and incubated for 30 min. The absorbance of the mixture was determined at 765 nm with a spectrophotometer. Gallic acid was used as a reference standard, and the results were expressed as mg gallic acid equivalents (GAE) per gram dry weight (DW) of MWE.

### 4.4. Separation and Identification of Flavonoids Using NMR 

To identify major flavonoids from MWE, we used high-performance liquid chromatography (HPLC) using a published method [35]. MWE dissolved with deionized water was subjected to HPLC equipped with a diode array detector (DAD) (Waters 600, Milford, MA, USA). The absorption of spectra was recorded from 210–600 nm for all peaks. UV absorbance at 280 nm and 337 nm was used to monitor phenolic compounds and flavonoids, respectively. An analytical column C18 (4.6 × 250 mm, 5µm, Waters Symmetry Column, Ireland) was used and kept at 30 °C. The injection volume of the sample was 10 µL. Elution was done using two solvent gradients: solvent A (1% acetic acid in deionized water, *v/v*) and solvent B (1% acetic acid in methanol, *v/v*). Elution was carried out at a flow rate of 1 mL/min. The gradient program was as follows: 10–35% B (10 min), 35–42% B (15 min), 42–75% B (10 min), 75% B (5 min), 75–10% B (5 min), and 10% B (5 min).

To further confirm whether the flavonoids were vitexin and isovitexin, we performed NMR analyses. First, we prepared a large batch size MWE and used semi-preparative HPLC to separate both flavonoids from MWE. The semi-preparative HPLC was carried out using a published method [35] with some modification using Shimadzu LC-20AP system equipped with a DAD (SPD-M20A, Shimadzu, Kyoto, Japan) and Inertsil ODS-3, C18 column (4.6 × 250, 5 µm, Waters symmetry, Wexford, Ireland). The injection volume of MWE was 1 mL. Elution was done using 40% methanol and 60% water. The eluates were carefully collected corresponding to the retention time of the two major peaks (referred to as fraction 1 and fraction 2). This isolation process was repeated 5 times to obtain a sufficient amount of the flavonoids for NMR analyses. The collected fractions were evaporated with a rotary evaporator (Buchi R-300, Flawil, Switzerland) at 100 rpm, 60 °C for 40–60 min, and freeze-dried for 20 h to obtain the lyophilized MWE (Christ, Gamma 2–16, LSC, Osterode, Germany). The collected fractions were then determined for their purities using HPLC equipped with DAD with the above chromatographic condition. The purity of the collected fractions 1 and 2 was more than 99%.

The lyophilized samples were carefully dissolved in DMSO-*d_6_* to elucidate their structures using ^1^H-NMR and ^13^C-NMR spectrometer (Ascend TM 600, Bruker, France).

### 4.5. Standardization of Major Flavonoids in MWE

Vitexin and isovitexin were dissolved in dimethyl sulfoxide (DMSO) and diluted to get the concentration 5–50 mg/L to make standard curves using HPLC-DAD with a previous condition. The concentrations of vitexin and isovitexin in MWE were calculated according to the standard curves. The concentrations of vitexin and isovitexin in each replication of MWE were standardized before further analysis.

### 4.6. Assay of A-Amylase Inhibition

Inhibitory effects of MWE against α-amylase were determined according to the spectrophotometric method [5] with some modifications. Briefly, 125 µL of MWE and 125 µL of α-amylase (2 units/mL) were mixed and pre-incubated at 37 °C for 5 min. Then, 250 µL of 0.2% (*w/v*) starch dissolved in the deionized water was added to the reaction mixture to make a total volume of 500 µL, and the whole mixture was incubated at 37 °C for 5 min. After the incubation, 250 µL of dinitrosalicylic acid (DNS) color reagent was added and placed in a boiling bath for exactly 5 min to stop the reaction. Then, this mixture was cooled on ice to room temperature. The α-amylase activity was determined by measuring the absorbance of the mixture at 540 nm by a microplate reader (Tecan Infinite 200 Pro, Männedorf, Switzerland). Control was conducted in an identical fashion replacing MWE with deionized water. For blank, the enzyme solution was replaced with deionized water, and the same procedure was carried out as above. Acarbose was used as a positive control. The results were reported as % relative activity. The concentration of MWE required to inhibit the enzyme activity by 50% (IC_50_) was determined using the GraphPad Prism program.

### 4.7. Assay of α-Glucosidase Inhibition

The inhibitory effect of MWE against α-glucosidase was determined with the spectrophotometric method [36]. Briefly, 20 µL of MWE and 20 µL of α-glucosidase (1 unit/mL) were mixed and pre-incubated in 460 µL of 0.1 M potassium phosphate buffer solution (pH 6.8) at 37 °C for 20 min. Then, 200 µL of 1 mM pNPG was added to initiate the reaction. The mixture was further incubated at 37 °C for 15 min. The reaction was terminated by the addition of 500 µL of 1 M Na_2_CO_3_ in deionized water. The absorbance was spectrophotometrically measured at 405 nm (Tecan Infinite 200 Pro, Switzerland). Control was conducted in an identical fashion replacing MWE with deionized water. For blank, the enzyme solution was replaced with deionized water, and the same procedure was carried out as above. The result was expressed as % relative activity. Acarbose was used as a positive control. The IC_50_ value was determined using the GraphPad Prism program.

### 4.8. Inhibition of Advanced Glycation End Products (Ages) Formation Assay

The inhibitory effect of MWE against the formation of AGEs was evaluated by bovine serum albumin (BSA)-glucose assay [15] with some modifications. Briefly, BSA and D-glucose were dissolved in phosphate buffer (0.5 M, pH 7.4) to obtain a control solution with 50 mg/mL BSA and 0.8 M D-glucose. One milliliter of the control solution was incubated at 37 °C for 7 days in the presence or absence of 0.5 mL of MWE. The test solution also contained NaN_3_ (0.02%) to assure an aseptic condition. Aminoguanidine (AG) was used as a positive control. After a 7-day incubation, fluorescence intensity was measured at excitation wavelength 330 nm and emission wavelength 410 nm (Tecan Infinite 200 Pro, Switzerland). The percentage of AGEs formation was calculated and expressed as % relative formation. The IC_50_ value was determined using the GraphPad Prism program.

### 4.9. Protein Tyrosine Phosphatase 1B (PTP-1B) Inhibition Assay 

The PTP-1B inhibition assay was determined according to the previous method [33]. Briefly, 10 µL of MWE was mixed and pre-incubated with 40 µL of PTP-1B (1.25 µg/mL) at 37 °C for 10 min. Fifty microliters of pNPP was added to initiate the reaction and further incubated at 37 °C for 30 min. The reaction was quenched by the addition of 20 µL of 10 M NaOH. Dephosphorylation of pNPP generates pNP, which was spectrophotometrically monitored at 405 nm (Tecan Infinite 200 Pro, Switzerland). NaVO_4_ was used as a positive control. The result was expressed as % relative activity. The IC_50_ value was determined using the GraphPad Prism program.

### 4.10. Cell culture and Cytotoxicity Assay

HepG2 cells (human hepatoma cell line) were purchased from American Type Culture Collection (ATCC, Rockville, MD, USA). HepG2 cells were cultured in Dulbecco’s modified Eagle’s medium (DMEM) (1 g/L glucose) supplemented with 10% fetal bovine serum, 100 units/mL of penicillin, 100 units/mL of streptomycin, and 1% of non-essential amino acid) in a humidified atmosphere in 5% CO_2_ at 37 °C. The medium was changed every 2 days and subcultured once the cells reached 90% confluence. 

To determine cytotoxicity, HepG2 cells were seeded at the density of 2 × 10^4^ cells/well in a 96-well plate. After 24 h culture, the medium was changed and incubated with different concentrations of MWE (0.5–20 mg/mL) for 24h. Cytotoxicity of MWE on HepG2 cell viability was assessed by 3-(4,5-dimethylthiazol-2-yl)-2,5-diphenyltetrazolium bromide (MTT) assay. Briefly, at the end of the incubation time, 10 μL of 5 mg/mL of MTT was added into each well and incubated for 4 h. After that, MTT-containing medium was gently removed and replaced with 100 μL DMSO to dissolve the formazan crystals. The plate was gently shaken for 15 min so that complete dissolution was achieved. Cell viability was observed by recording the absorbance at 570 nm (TECAN, Infinite 200 PRO, Switzerland). Results were presented as percentages of the control values [33].

### 4.11. Cellular Glucose Uptake Assay

Cellular glucose uptake was quantified by the 2-deoxy-2-[(7-nitro-2,1,3-benzoxadiazol-4-yl)amino]-D-glucose (2-NBDG) assay described by Zou et al. [37]. Briefly, HepG2 cells were seeded at the density of 2 × 10^4^ cells/well in a 96-well fluorescence plate. After 24 h culture, the medium was changed and incubated with serum-free medium with 1 μM insulin for 24 h to induce insulin resistance. Then, the medium was replaced with different concentrations of MWE in a serum-free medium and incubated for another 24 h. After 24 h incubation, serum-free medium containing MWE was replaced with 100 nM insulin in PBS and incubated for 30 min, then 10 μL of 2-NBDG (40 μM in PBS) was added and incubated for 30 min. The cells were washed three times with ice-cold PBS. Fluorescence intensity was immediately measured at an excitation wavelength of 460 nm and an emission wavelength of 528 nm. An estimation of the overall glucose uptake was obtained by quantifying the fluorescence. The percentage of cellular glucose uptake was calculated using the following equation:

% cellular glucose uptake = (fluorescence intensity of cells treated with MWE/fluorescence intensity of the control cells) × 100%

The fluorescent 2-NBDG images were determined by treating the cells in Lab-Tek 8 wells chamber slide with the same protocol above. After the cells were incubated with 2-NBDG for 30 min, the cells were washed three times with ice-cold PBS. The chamber was removed, and the slide was covered with a cover slide. The retain fluorescence was detected by a fluorescence microscope (Zeiss, Axio A1) and analyzed with Image-Pro Plus 6.0 program.

### 4.12. Gene Expression of Insulin-Resistant Hepg2 Cells 

For RNA harvesting, HepG2 cells were seeded at the density of 3 × 10^5^ cells/well in a 12-well plate. Total RNA was isolated using the GeneJET RNA Purification Kit (Thermo Scientific, Vilnius, Lithuania). DNA was removed from RNA samples by using DNase I, RNase-free (Thermo Scientific, Lithuania). The concentration of RNA was quantified using NanoDrop Spectrophotometer. Complementary DNA (cDNA) was synthesized from isolated RNA with RevertAid First Strand cDNA Synthesis Kit (Thermofisher Scientific, Lithuania). Quantitative real-time PCR (qRT-PCR) analysis was performed by the SYBR Green method using SYBR Green Supermix (Bio-rad) on Bio-Rad System with CFX Manager Software. The PCR primer sequences used were as follows: FOXO1, forward 5′-TTCACCCAGCCCAAACTACC-3′, and reverse 5′-GAGTCCAGGCGCACAGTTAT-3′; PEPCK, forward 5′- CTTTGGAGGCCGTAGACCTG-3′, and reverse 5′- GCCTTTATGTTCTGCAGCCG-3′; GSK-3β forward 5′-TCGTCCATCGATGTGTGGTC-3′, and reverse 5′-TTGTCCAGGGGTGAGCTTTG-3′; GAPDH, forward 5′-ACGACCAAATCCGTTGACTC-3′, and reverse 5′-CTCTCTGCTCCTCCTGTTCG-3′. Ct values were normalized to GAPDH and the relative gene expression was calculated with 2^−ΔΔCt^ method.

### 4.13. Statistical Analysis

All data were expressed as mean ± standard deviation and were done in triplicate independent analyses. The results obtained were analyzed by SPSS version 23.0 (IBM Software, Armonk, NY, USA). Any significant difference was determined by one-way analysis of variance (ANOVA) followed by Duncan’s multiple range test for multiple comparisons at *p* < 0.05 level. GraphPad Prism 7 statistical package (GraphPad Software Inc, La Jolla, CA 92037, USA) was used to determine IC_50_ values. 

## 5. Conclusions 

This is the first study to explore the anti-diabetic effects of mungbean extracted using boiling water. Using non-organic solvent in the extraction is considered safe for human consumption, practical large-scale preparation, and a sustainable environmental manufacturing process. The HPLC and NMR analyses revealed that MWE contained both isomeric flavonoids vitexin and isovitexin and exerted anti-diabetic effects. MWE inhibited α-amylase, α-glucosidase, AGEs formation, and PTP-1B. In the insulin-resistant HepG2 cells, MWE improved insulin signaling, resulting in the increase of cellular glucose uptake and suppressed mRNA expression of FOXO1, PEPCK, and GSK-3β. Our results suggested that MWE containing vitexin and isovitexin can alleviate insulin resistance relevant to PTP-1B inhibition. Further investigation is required to support the use of mungbean as a functional food to improve insulin resistance conditions in diabetes. 

## Data Availability

Supplementary materials are available online: Figures S1–S3.

## Supplementary Materials

The following are available online. Figure S1: Representative HPLC-DAD chromatographic profiles of (A) MWE, (B) standard vitexin 50 μg/mL, and (C) standard isovitexin 50 μg/mL (λ = 337 nm). Figure S2: Inhibitory activities of acarbose on (A) α-amylase and (B) α-glucosidase. Values are expressed as mean ± SD (n = 3). Figure S3: Inhibitory activities of (A) aminoguanidine (AG) on AGEs formation and (B) NaVO_4_ on PTP-1B activity. Values are expressed as the mean ± SD (n = 3).

## Sample Availability

Samples of the compounds are not available from the authors.

## Abbreviations

ACE	angiotensin I-converting enzyme
AG	aminoguanidine
AGEs	advanced glycation end products
ANOVA	analysis of variance
AU	arbitrary unit
BSA	bovine serum albumin
DAD	diode array detector
DMEM	Dulbecco’s modified Eagle’s medium
DMSO-d_6_	deuterated dimethylsulfoxide
DNS	dinitrosalicylic acid
DW	dry weight
EGCG	(-)-epigallocatechin-3-gallate
FOXO1	forkhead box O1
GAPDH	glyceraldehyde 3-phosphate dehydrogenase
GAE	gallic acid equivalent
GSK-3β	glycogene synthase kinase-3β
HPLC	high-performance liquid chromatography
IR	insulin-resistant HepG2 cells
MWE	mungbean water extract
MTT	3-(4,5-dimethylthiazol-2-yl)-2,5-diphenyltetra-zolium bromide
NaN_3_	sodium azide
2-NBDG	2-deoxy-2-[(7-nitro-2,1,3-benzoxadiazol-4-yl)amino]-D-glucose
NMR	nuclear magnetic resonance
PCR	polymerase chain reaction
PEPCK	phosphoenolpyruvate carboxykinase
PI-3K	phosphatidylinosital 3-kinase
PKB/Akt	protein kinase B
pNP	p-nitrophenyl
pNPG	para-nirophenyl-α-d-glucopyranoside
pNPP	p-nitrophenyl phosphate
PTP-1B	protein tyrosine phosphatase-1B
UV	ultraviolet
